# LiCoO_2_ particles used in Li-ion batteries induce primary mutagenicity in lung cells via their capacity to generate hydroxyl radicals

**DOI:** 10.1186/s12989-020-0338-9

**Published:** 2020-01-29

**Authors:** Violaine Sironval, Vittoria Scagliarini, Sivakumar Murugadoss, Maura Tomatis, Yousof Yakoub, Francesco Turci, Peter Hoet, Dominique Lison, Sybille van den Brule

**Affiliations:** 1 Louvain centre for Toxicology and Applied Pharmacology, Institut de Recherche Expérimentale et Clinique, Université catholique de Louvain, Avenue Hippocrate 57, box B1.57.06, 1200 Brussels, Belgium; 2 Laboratory of Toxicology, Unit of Environment and Health, Department of Public Health and Primary Care, Katholieke Universiteit Leuven, Herestraat 49 - O&N1 - Room: 07.702, box 706, 3000 Leuven, Belgium; 3 Department of Chemistry and “G. Scansetti” Interdepartmental Center for Studies on Asbestos and Other Toxic Particulates, University of Torino, Via P. Giuria 7, 10125 Torino, Italy

**Keywords:** Genotoxicity, Micronucleus assay, Comet assay

## Abstract

**Background:**

Li-ion batteries (LIB) are used in most portable electronics. Among a wide variety of materials, LiCoO_2_ (LCO) is one of the most used for the cathode of LIB. LCO particles induce oxidative stress in mouse lungs due to their Co content, and have a strong inflammatory potential. In this study, we assessed the mutagenic potential of LCO particles in lung cells in comparison to another particulate material used in LIB, LTO (Li_4_Ti_5_O_12_), which has a low inflammatory potential compared to LCO particles.

**Results:**

We assessed the mutagenic potential of LCO and LTO particles in vitro by performing a cytokinesis-block micronucleus (MN) assay with rat lung epithelial cells (RLE), as well as in vivo in alveolar type II epithelial (AT-II) cells. LCO particles induced MN in vitro at non-cytotoxic concentrations and in vivo at non-inflammatory doses, indicating a primary genotoxic mechanism. LTO particles did not induce MN. Electron paramagnetic resonance and terephthalate assays showed that LCO particles produce hydroxyl radicals (•OH). Catalase inhibits this •OH production. In an alkaline comet assay with the oxidative DNA damage repair enzyme human 8-oxoguanine DNA glycosylase 1, LCO particles induced DNA strand breaks and oxidative lesions. The addition of catalase reduced the frequency of MN induced by LCO particles in vitro.

**Conclusions:**

We report the mutagenic activity of LCO particles used in LIB in vitro and in vivo. Our data support the role of Co(II) ions released from these particles in their primary genotoxic activity which includes the formation of •OH by a Fenton-like reaction, oxidative DNA lesions and strand breaks, thus leading to chromosomal breaks and the formation of MN. Documenting the genotoxic potential of the other LIB particles, especially those containing Co and/or Ni, is therefore needed to guarantee a safe and sustainable development of LIB.

## Background

Li-ion batteries (LIB) are used in most portable electronics. This technology has replaced nickel-cadmium and nickel metal hydride batteries because of its higher energy density, higher efficiency and longer life. Low weight, design flexibility and size are other advantages of LIB [1, 2]. The LIB anode usually consists of porous carbon, and the cathode is made of Li metal oxide particles. As these particles are respirable in size, poorly soluble and persist in the lung, the health risks associated with human exposure should be carefully evaluated, especially in occupational settings. Moreover, future applications of LIB, such as multi-layer systems made for spray-paintable or printable DIY batteries [3–5], might extend the potential for inhalation exposure to consumers. LiCoO_2_ (LCO) particles are one of the most used cathode material for LIB [6]. We showed in recent experimental studies that LCO particles induce lung oxidative stress, inflammation, and fibrosis in mice [7, 8]. The mutagenic and carcinogenic potential of LCO particles has not been examined yet.

The genotoxic potential of inhaled particles is defined by their ability to induce DNA damage via a primary and/or a secondary mechanism. Primary genotoxicity is due to the intrinsic characteristics of the particles, including composition, shape, size, crystallinity or their capacity to produce reactive oxygen species (ROS). Secondary genotoxicity is associated with the production of ROS by leukocytes recruited during lung inflammation induced by the inhalation of these particles [9]. Mutations occur when DNA damage is not (well) repaired and persists after cell division. Several inhaled particles or fibres have a mutagenic activity, including crystalline silica via a secondary mechanism [10] or asbestos via primary and secondary mechanisms [11]. Assessing the genotoxicity and mutagenic activity of LCO particles appears, therefore, relevant as these particles have a strong inflammatory potential, even stronger than crystalline silica particles, and induce oxidative stress in mouse lungs [7]. Moreover, LCO particles contain bioaccessible cobalt [7, 8]. Co(II) ions have a genotoxic activity due to their ability (i) to produce hydroxyl radicals (•OH) via a Fenton-like reaction and (ii) to interact with and inhibit proteins, including those implicated in DNA repair [12]. In 2006, the International Agency for Research on Cancer (IARC) classified cobalt sulfate, other soluble cobalt(II) salts and cobalt metal as possibly carcinogenic to humans (Group 2B) and cobalt metal with tungsten carbide (WC-Co) as probably carcinogenic to humans (Group 2A) [13]. In this paper, we assess the mutagenic potential of LCO particles, and related mechanisms, in comparison with another particulate material used in LIB, LTO (Li_4_Ti_5_O_12_) which does not contain genotoxic metals and has a low inflammatory potential compared to LCO [7].

## Results

### LCO particles induce micronuclei in lung epithelial cells in vitro

**Fig. 1:**
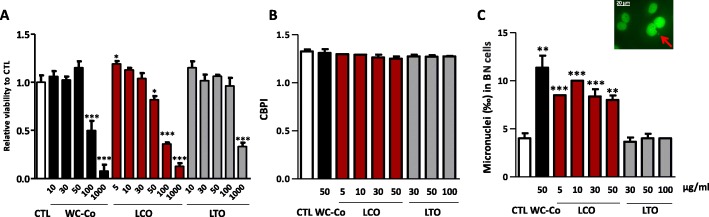
LCO particles induce MN in lung epithelial cells in vitro. Rat lung epithelial cells (RLE, 55556 cells/cm^2^) were exposed to culture medium (control, CTL), WC-Co, LCO or LTO, and cytotoxicity was assessed after 24 h by the WST-1 assay (**a**). The CBPI (**b**) was assessed in 500 cells exposed to non-cytotoxic particle concentrations, and the frequency of MN determined in 1000 binucleated cells (**c**). Image of a binucleated cell containing a micronucleus designated by the red arrow (**c**). *P < 0.05, **P < 0.01 and ***P < 0.001 relative to CTL cells (t-test or one-way ANOVA followed by a Dunnett’s multiple comparison). Bars represent means ± SEM (*N* = 2 for results obtained with 5 μg/ml LCO; *N* = 4 for all other results with *n* = 2 for CTL and *n* = 4 for all other conditions)

Within the framework of the 3R’s (Replacement, Reduction and Refinement) strategy proposed by the European legislation [14], we first assessed the mutagenic activity of LCO particles in vitro by using the cytokinesis-block micronucleus (MN) assay on rat lung epithelial cells (RLE) [15]. WC-Co particles were used as positive control. We first determined non-cytotoxic concentrations. After 24 h, WC-Co was non-cytotoxic up to 50 μg/ml, LCO was non-cytotoxic up to 30 μg/ml and very weakly cytotoxic at 50 μg/ml, and LTO was non-cytotoxic up to 100 μg/ml (Fig. 1a). Fifty μg/ml WC-Co, 5–50 μg/ml LCO and 30–100 μg/ml LTO were selected to perform the cytokinesis-block MN assay.

We next performed the cytokinesis-block MN assay. The proliferation of RLE (assessed by the cytokinesis-block proliferation index, CBPI) was not significantly altered by the particles at these concentrations (Fig. 1b, LCO: ANOVA *p* = 0.6307, trend test *p* = 0.2337, LTO: ANOVA *p* = 0.9754, trend test *p* = 0.8676). Like WC-Co, LCO particles increased MN frequency at all concentrations tested, indicating a primary mutagenic activity (Fig. 1c). LTO particles did not increase MN frequency. To assess the influence of endocytosis on our results (cytochalasin B used to block cytokinesis can inhibit endocytosis), we counted binucleated cells containing particles in their cytoplasm and the number of particles per binucleated cells. Particles were visible in approximately 80% of the binucleated cells 24 h after treatment and this proportion, as well as the number of particles per binucleated cells, were similar for both LCO or LTO particles (see Additional file 1: Figure S1).

### LCO particles induce micronuclei in lung epithelial cells in vivo

**Fig. 2:**
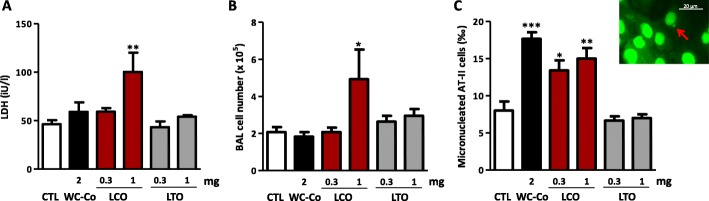
LCO particles induce MN in lung epithelial cells in vivo. Wistar rats were treated with an oro-pharyngeal aspiration of NaCl (control, CTL), WC-Co, LCO or LTO particles. Inflammation and MN were assessed after 3 d. LDH activity (**a**) was measured in the BALF, recruited inflammatory cells (**b**) in the BAL and the frequency of micronuclei (**c**) in AT-II cells isolated from rat lungs. Image of an AT-II cell containing a micronucleus designated by the red arrow (**c**). *P < 0.05, **P < 0.01 and ***P < 0.001 relative to CTL mice (t-test or one-way ANOVA followed by a Dunnett’s multiple comparison). Bars represent means ± SEM (*N* = 2, *n* = 4 for the first experiment and *n* = 2 for the second experiment)

We next confirmed the mutagenic activity of LCO particles in vivo*,* as proposed by the REACH regulation [16], using a MN assay in isolated rat alveolar type II epithelial (AT-II) cells. To determine non-inflammatory and inflammatory doses, rats were first treated with an oro-pharyngeal aspiration of 0.1, 0.3, 1 or 5 mg of LCO or LTO particles. Lactate dehydrogenase (LDH) activity (a marker of cytotoxicity), protein concentrations (a marker of alveolar permeability) and inflammatory alveolar cell infiltration were measured in broncho-alveolar lavage (BAL) 3 d after administration (See Additional file 1: Figure S2). Based on these results, doses of 0.3 and 1 mg LCO were selected for the MN assay as non-inflammatory and inflammatory doses, respectively, to help to discriminate mutations due to primary and secondary genotoxic mechanisms (Fig. 2a, b). WC-Co was used as positive control at the dose of 2 mg [17]. The frequency of MN was assessed in rat lung AT-II cells isolated 3 d after administration of the particles (Fig. 2). This time point captures the impact of acute inflammation [7, 18], and allows AT-II cells to undergo in vivo division and to reveal MN [17]. As expected, increased MN frequencies were detected after WC-Co (Fig. 2c). LCO particles also increased MN frequencies at the doses of 0.3 and 1 mg, confirming that they act, at least, via a mechanism of primary genotoxicity. LTO particles did not increase MN frequency in vivo.

### LCO particles have an intrinsic capacity to generate hydroxyl radicals

**Fig. 3:**
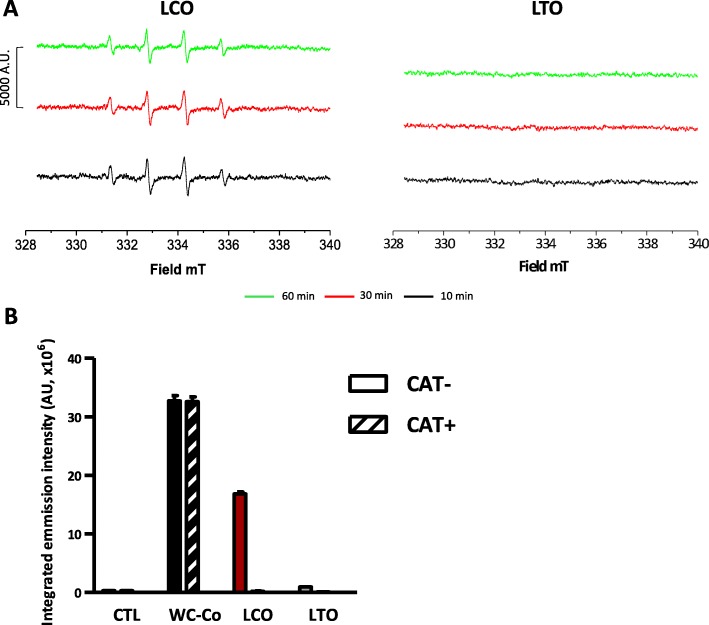
Generation of hydroxyl radicals by LCO particles. EPR spectra (**a**) of [DMPO-HO]• adducts after incubation with 25 mg/ml LCO or LTO particles in the presence of H_2_O_2_ (0.2 M) under gentle agitation. Spectra were collected after 10, 30 and 60 min. Fluorescence intensity (**b**) recorded on the supernatant from 5 mg/ml WC-Co, LTO or LCO particles incubated 15 min (for WC-Co) or 30 min (for LCO and LTO) in a PBS solution of disodium TA (10 mM) with H_2_O_2_ (0.2 M) under gentle agitation, in absence (CAT-) or in presence of 3000 U/ml catalase (CAT+). Control (CTL) did not contain particles (*N* = 2, *n* = 4 for the control condition and *n* = 6 for all other conditions)

Because of their cobalt content, we investigated the capacity of LCO particles to produce •OH by using an electron paramagnetic resonance (EPR) assay (Fig. 3a). LCO particles constantly produced •OH over 60 min. No •OH production was observed with LTO particles (Fig. 3a). As •OH are the most potent DNA interacting ROS and can induce DNA breaks [19], they could account for the primary genotoxic activity of LCO particles.

### LCO particles induce oxidative DNA damage in RLE in vitro

**Fig. 4:**
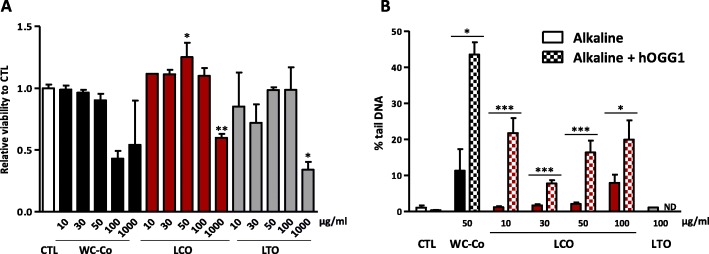
LCO particles induce DNA strand breaks and DNA oxidative lesions in lung epithelial cells in vitro. Rat lung epithelial cells (RLE, 15600 cells/cm^2^) were exposed to culture medium (control, CTL), WC-Co, LCO or LTO and cytotoxicity was assessed after 24 h by the WST-1 assay (**a**). Alkaline comet assay, with or without oxidative DNA lesion repair enzyme (hOGG1), was performed 24 h after exposure to particles (**b**). *P < 0.05, **P < 0.01 and ***P < 0.001 (t-test between alkaline and alkaline + hOGG1 conditions). Bars represent means ± SEM (*N* = 4 with *n* = 4 for the WST-1 assay, *N* = 4 for the alkaline comet assay performed without hOGG1 and *N* = 2 for the alkaline comet assay performed with hOGG1, *n* = 2). ND = not determined

To further investigate whether •OH produced by LCO particles contribute to their genotoxic activity, we applied the comet assay in the presence of the oxidative DNA damage repair enzyme human 8- oxoguanine DNA glycosylase 1 (hOGG1). hOGG1 specifically recognizes and cleaves oxidative lesions leading to additional DNA fragments. We first assessed the cytotoxicity of particles on RLE (Fig. 4a) in the culture conditions used for the comet assay. RLE were exposed to 10–1000 μg/ml WC-Co, 10–1000 μg/ml LCO or LTO particles during 24 h. After 24 h, WC-Co was non-cytotoxic up to 50 μg/ml and LCO and LTO up to 100 μg/ml (Fig. 4a). Fifty μg/ml WC-Co, 10–100 μg/ml LCO and 100 μg/ml LTO were used to perform the comet assay. As expected, WC-Co induced DNA strand breaks and oxidative lesions as the % tail DNA increased when cells were treated with hOGG1 (Fig. 4b) [20]. DNA strand breaks were induced in a dose-dependent manner by LCO particles. The addition of hOGG1 revealed additional DNA breaks, reflecting the presence of oxidative lesions. LTO particles did not induce DNA breaks (Fig. 4b). The same results were obtained with another oxidative damage repair enzyme, the *E. coli* formamidopyrimidine-DNA glycosylase (FPG, data not shown).

### Catalase prevents the formation of MN by LCO particles

To assess the implication of oxidative DNA lesions in the induction of MN by LCO particles, we used catalase to block the formation of •OH in the Fenton-like reaction. We first performed a terephthalate (TA) assay with catalase to verify its capacity to inhibit •OH formation in our system. LCO, LTO or WC-Co particles were incubated in TA with or without catalase during 15 or 30 min. Addition of catalase prevented •OH production by LCO particles (Fig. 3b). LTO particles did not generate •OH in this test. As expected, •OH produced by WC-Co were not affected by catalase as •OH produced by WC-Co are independent of the presence of H_2_O_2_ [21].

**Fig. 5:**
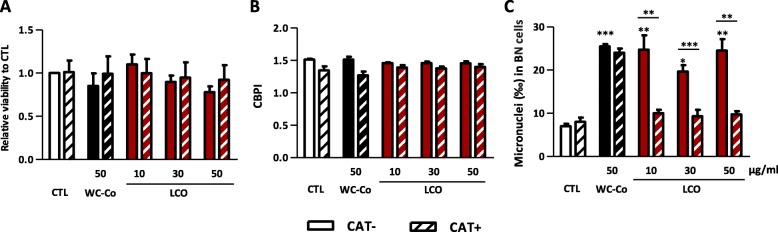
LCO particles induces MN via •OH generation. Rat lung epithelial cells (RLE, 55556 cells/cm^2^) were exposed to culture medium (control, CTL), WC-Co, LCO particles in absence (CAT-) or in presence of 3000 U/ml catalase (CAT+). Cytotoxicity was assessed after 24 h by the CellTiter-Glo Luminescence viability test (**a**). The CBPI (**b**) was assessed in 500 cells, and the number of MN in 1000 binucleated cells (**c**). *P < 0.05, **P < 0.01 and ***P < 0.001 (t-test or one-way ANOVA followed by a Dunnett’s multiple comparison relative to the control condition, and t-test between CAT- et CAT+ conditions). Bars represent means ± SEM (*N* = 2 for cytotoxicity assessment and *N* = 3 for CPBI and MN assessment, *n* = 4 for cytotoxicity assessment and *n* = 2 for CBPI and MN assessment)

We next performed the cytokinesis-block MN assay in RLE with catalase (Fig. 5). RLE were exposed to 50 μg/ml WC-Co, or 10–50 μg/ml LCO particles with or without catalase. This assay was not conducted with LTO particles as they did not induce MN (Fig. 1). Twenty-four h after particle exposure without catalase, cell viability and proliferation was not affected by the particles (Fig. 5a, b). For this experiment, we performed the CellTiter-Glo Luminescent viability assay to avoid possible interference between the yellow color of catalase and the colorimetric WST-1 assay used in previous experiment (Fig. 1a). Addition of catalase did not affect cell viability or proliferation. Catalase did not modify the mutagenic potential of WC-Co particles (Fig. 5c) as expected (Fig. 3). In contrast, MN induced by LCO particles were less frequent in presence of catalase, indicating that •OH produced by LCO particles contribute to the formation of MN.

## Discussion

We demonstrate here the primary mutagenic activity of LCO particles used in LIB. These particles are able to induce mutations in vitro and in vivo, while LTO particles do not appear genotoxic.

We selected the MN assay to assess the genotoxic potential of these particles because this test detects mutations relevant for the carcinogenic process [15]. Advantages of the MN assay compared to other mutagenicity tests are its capacity to detect both clastogenic and aneugenic events, and the epidemiological evidence of its predictive value in terms of cancer risk [11, 22].

LCO particles induced MN in AT-II cells isolated from rat lungs at a non-inflammatory dose indicating that they can act in the lung via a primary genotoxic mechanism. LCO particles also induced a slightly higher MN frequency at the inflammatory dose reflecting either a secondary mechanism of genotoxicity or a dose-dependent primary effect. The primary genotoxic activity of LCO particles was also observed in vitro where the use of cytochalasin B allowed controlling any confounding of altered cell division or cytotoxicity induced by the particles [15]. In the in vitro assay, the formation of MN was not dose-dependent, suggesting a maximum of MN induction at the lowest concentration, or a slight cytotoxicity not detected by the CBPI.

LCO particles contain bioaccessible Co [7]. We suspected cobalt ions and their capacity to produce •OH [12] to be involved in the mutagenic activity of LCO particles. ROS are implicated in the genotoxic activity of several inhaled particles. They can attack DNA and lead to base pair mutations, deletions or insertions, and induce DNA strand breaks. Two types of ROS may be generated, (i) ROS intrinsically generated by particles and (ii) ROS produced by inflammatory and/or target cells in response to particles [19]. We assessed the capacity of LCO particles to intrinsically produce ROS by the EPR and TA assays. H_2_O_2_ was included to mimic the reaction that might occur in the lysosomes of macrophages or polymorphonucleated cells, or in lung epithelial cells interacting with inhaled particles [23, 24]. Both assays showed that, unlike LTO, LCO particles produced •OH probably via a Fenton-like reaction which occurs between transition metal ions and H_2_O_2_ [25, 26]. LCO particles formally contain Co(III), but it has been previously shown that both Co(II) and Co(III) ions can be present at the particle surface [27]. In acidic conditions, Co(II) is the most stable oxidation state and Co(III) is rapidly reduced to Co(II) [28]. Both Co species can participate in their ionic form to a Fenton-like reaction by reacting with OOH^**−**^ deriving from H_2_O_2_ or directly with H_2_O_2_ [29]. •OH is the most potent ROS to interact with DNA and is a crucial factor in the clastogenic activity of inhaled particles [19]. In the in vitro alkaline comet assay with addition of the oxidative DNA damage repair enzymes, LCO particles induced oxidative DNA lesions, suggesting that •OH contribute to their primary genotoxic activity. The blocking effect of catalase supports this hypothesis. For particles, direct DNA damage requires their localization in the nucleus to interact with DNA [30]. Here, DNA damage seemed to be mainly mediated by the production of •OH, thus via an indirect mechanism, indicating that particle localization is not determinant in their genotoxic activity. On the other hand, Ortega et al. [31] showed that Co ions released from low soluble Co nanoparticles (Co_3_O_4_) can be found in the cytoplasm and the nucleus of epithelial cells, suggesting that the Fenton-like reaction induced by LCO Co(II/III) ions could occur in both cellular compartments.

Thus, these results indicate that LCO particles should be considered as presenting a carcinogenic hazard in case of inhalation since they exhibit 3 key characteristics of human carcinogens identified by Smith et al. [32]: the capacity to induce lung oxidative stress, and chronic inflammation [7], and a mutagenic activity. The capacity of LCO particles to release Co(II) ions appears responsible for their mutagenic activity.

In our previous study on a panel of LIB particles (LCO, LTO, LiNiCoAlO_2_, LiNiCoMnO_2_ and LiFePO_4_), we showed that particles containing Co and/or Ni can cause lung inflammation and fibrosis in mice [7, 8]. Since Ni compounds can also exert a mutagenic activity [33], other LIB particles containing Co and/or Ni could also be mutagenic. In addition, LCO and other LIB particles containing Co and/or Ni, strongly stabilize hypoxia-inducible factor (HIF) -1α in lung tissue [8], a transcription factor involved in tumor growth, angiogenesis and metastasis [34], further suggesting a potential carcinogenic activity of these particles.

## Conclusions

We established the primary mutagenic activity of LCO particles used in LIB in vitro and in vivo. Our data support the role of Co(II) ions released from these particles in their mechanism of mutagenicity, which includes the formation of •OH by a Fenton-like reaction and oxidative DNA lesions, thus leading to chromosomal breaks and the formation of MN. Documenting the genotoxic potential of the other particles containing Co/Ni used in LIB is needed to guarantee a safe and sustainable development of LIB.

## Methods

### Particles

LTO (Li_4_Ti_5_O_12_) and LCO (LiCoO_2_) particles were obtained from MTI Corporation (Richmond, USA), WC-Co from Metron (USA). Before all experiments (including characterization), particles were heated during 2 h at 200 °C to inactivate any possible endotoxin or other microbial contaminants. The physico-chemical characterization of heat-treated LTO and LCO particles was reported previously [7]. Particles were suspended in complete culture medium (in vitro assays) or 0.9% saline solution (in vivo experiments) without any further treatment.

### Epithelial cell culture

RLE cells (rat alveolar epithelial type II cells, RLE-6TN, doubling time > 30 h [35], ATCC, Virginia, USA) were cultured at 37 °C in complete medium, i.e. Ham’s F12 Nutrient Mix (Gibco, Paisley, UK) supplemented with 1% antibiotic-antimycotic (Gibco), 10% fetal bovine serum and 1% Glutamine (Gibco). Before exposure, RLE were plated in 96-well plates for assessing cell viability (55556 or 15600 cells/cm^2^), 24-well plates for comet assays (15600 cells/cm^2^), or Lab-Teck plates (55556 cells/cm^2^) for MN assays. After 24 h incubation in complete medium at 37 °C, cells were exposed to particles during 24 h in complete culture medium. For experiments inhibiting the formation of hydroxyl radicals, catalase (3000 U/ml, Sigma-Aldrich) was added to the cells with the particles.

### Cell viability assays

Cell viability was evaluated by using the water soluble tetrazolium salts (WST-1) assay (Roche, Mannheim, Germany, 5%) or the CellTiter-Glo Luminescent viability assay (Promega, USA) following manufacturer’s instructions.

### In vitro cytokinesis-block micronucleus assay

Four hours after the addition of particles to the cells, cytochalasin B was added (3 μg/ml, Sigma-Aldrich, Missouri, USA). After 24 h exposure, cells were washed twice with phosphate buffered saline (PBS), fixed 20 min in methanol and stained with acridine orange (0.012% in PBS). Five hundreds cells per well were counted with a Zeiss AxioImager fluorescence microscope (magnification × 400) for assessing the cytokinesis-block proliferation index (CBPI) [36, 37]:

$$ \mathrm{CBPI}=\frac{\mathrm{number}\ \mathrm{of}\ \mathrm{mononucleated}\ \mathrm{cells}+2\ \mathrm{x}\ \mathrm{number}\ \mathrm{of}\ \mathrm{binucleated}\ \mathrm{cells}+3\ \mathrm{x}\ \mathrm{number}\ \mathrm{of}\ \mathrm{multinucleated}\ \mathrm{cells}}{\mathrm{Total}\ \mathrm{number}\ \mathrm{of}\ } $$

One thousand binucleated cells per well were examined for the presence of 1, 2 or more MN following the criteria described previously [15].

### Particle endocytosis

We performed the in vitro cytokinesis-block micronucleus assay and examined the presence of particles in the cytoplasm of one hundred binucleated cells with a Zeiss AxioImager flurorescence microscope (magnification × 400).

### Animals and treatments

Female Wistar rats were purchased from Janvier Labs (St Bertevin, France). Eight-week-old animals were kept with sterile rodent feed and acidified water, and housed in positive-pressure air-conditioned units (25 °C, 50% relative humidity) on a 12 h light/dark cycle. LTO and LCO particles were suspended in sterile 0.9% saline solution and WC-Co in sterile H_2_O. Mice were randomly allocated to experimental groups. After anaesthesia with a mix of Nimatek, 7.5 mg/rat (Eurovet, Bladel, Nederland) and Rompun, 1.5 mg/rat (Bayer, Kiel, Germany) given intraperitoneally, 300 μl particle suspensions or NaCl (control groups) were directly administered by oro-pharyngeal aspiration. Rats were sacrificed 3 d after particle administration with an intraperitoneal injection of 30 mg sodium pentobarbital (Certa, Braine-l’Alleud, Belgium). Rats were sacrificed randomly.

### Assessment of the in vivo inflammatory responses

Broncho-alveolar lavage was performed by cannulating the trachea and infusing the lungs with 5 ml NaCl 0.9%. BAL was centrifuged 10 min at 4 °C (240 *g*). Cell-free supernatant (BALF) was used for biochemical measurements. After resuspension in NaCl, total BAL cells were counted in Turch (crystal violet 1%, acetic acid 3%). Lactate dehydrogenase (LDH) activity and total proteins were assayed on BALF (Cobas 8000, Roche Diagnostics).

### Ex vivo micronucleus assay on type II pneumocytes

The in vivo mutagenic potential of particles was evaluated on type II pneumocytes (AT-II cells) isolated 3 d after rat exposure as described previously [38]. Isolated cells (an average of 12 × 10^6^ ATII cells/rat) were cultured during 2 d at 37 °C and then fixed 20 min in methanol 100% and stained with acridine orange. Cells were then analyzed with a Zeiss AxioImager fluorescence microscope. One thousand AT-II cells per rat were evaluated for the presence of MN.

### Electron paramagnetic resonance/spin trapping

Twenty-five mg particles were incubated in 0.5 ml PBS (0.5 M, pH 7.4, Sigma-Aldrich), 0.25 ml 5,5-dimethyl-l-pyrroline-N-oxide (DMPO, 0.15 M, Alexis, Lausen, Switzerland) used as spin-trapping agent and 0.25 ml H_2_O_2_ (0.2 M, Sigma-Aldrich) in order to analyse the •OH radical production. Particle suspensions were incubated under gentle agitation. Aliquots of 50 μl were withdrawn after 10, 30 and 60 min of incubation, filtered to remove particles and the generation of free radicals was monitored by EPR spectroscopy with a Miniscope MS 100 (Magnettech, Berlin, Germany) EPR spectrometer. The instrument settings were as follows: microwave power 10 mW, modulation 1000 mG, scan range 120 G, center of field approximately 3345 G.

### Sodium terephthalate (TA) assay

Particles (5 mg/ml) were suspended in TA solution (10 mM in PBS, pH 7.4) supplemented with H_2_O_2_ (0.2 M) and incubated 30 min (for LTO and LCO) or 15 min (for WC-Co) under gentle agitation at 25 °C [39]. To inhibit hydroxyl radical formation, catalase was added (3000 U/ml). After incubation, solutions were filtered (Millex-GS sterile filter Unit with MF-Millipore MCE membrane, 0.22 μm, Merck, Darmstadt, Germany). Fluorescence was measured with SpectraMax (excitation light = 324 nm, emission light = 425 nm).

### Comet assay (single-cell gel electrophoresis)

The DNA strand breaks induced by particles after 24 h were assessed in RLE cells by using an alkaline comet assay (Trevigen, Kampenhout, Belgium) [40] following manufacturer’s instructions. The analyses of oxidative DNA damage were performed by using comet assay in conjunction with *E. coli* formanidopyrimidine-DNA glycolase (FPG) and human 8-oxoguanine DNA glycosylase 1 (hOGG1) (Trevigen). Results were analyzed with a Zeiss AxioImager fluorescence microscope (magnification × 100) as described in the OECD test guidelines 489. Fifty cells from 2 replicates were measured for DNA damage by means of the % DNA tail metric using the CaspLab program (casplab 1.2.3b2) according to the following formula:

$$ \mathrm{DNA}\ \mathrm{tail}\ \left(\%\right)=\frac{\mathrm{Tail}\ }{\mathrm{Head}+\mathrm{Tail}}\mathrm{x}\ 100 $$

The means of the two medians for each condition were represented (OECD test guidelines 489).

### Statistics

Graphs and statistical analyses were performed with GraphPad Prism 5.0. All results are expressed as means ± standard error on the mean (SEM of N independent experiments, each conducted with n replicates). Differences between control and treated groups were evaluated by one-way analysis of variance (ANOVA) followed by a Dunnett’s multiple comparison or a t-test as appropriate. Statistical significance was considered at *P* < 0.05.

## Supplementary information

**Additional file 1: Figure S1.** LCO and LTO particle endocytosis by RLE cells. Rat lung epithelial cells (RLE) were exposed to NaCl or 50 μg/ml of LCO or LTO and treated with cytochalasin B 4 h after particle exposure. Endocytosis was assessed 24 h after particle exposure. Images of binucleated cells after treatment. White arrows designate some particles **(a)**. One hundred binucleated cells were scored to determine the proportion of cells containing particles **(b)** as well as the number of endocytosed particles per cell **(c)**. Bars represent means ± SEM (*N* = 1, *n* = 2). **Figure S2.** Inflammatory dose-response to LCO and LTO particles. Wistar rats were treated with an oro-pharyngeal aspiration of NaCl (control, CTL), 0.1, 0.3, 1 or 5 mg LCO or LTO particles. Inflammation was assessed after 3 d. LDH activity **(a)** and proteins **(b)** were measured in the BALF, recruited inflammatory cells in the BAL **(c)**. Bars represent means ± SEM. *P < 0.05, **P < 0.01 and ***P < 0.001 relative to CTL mice (one-way ANOVA followed by a Dunnett’s multiple comparison, N = 1, *n* = 4).

## Data Availability

The datasets used and/or analyzed during the current study are available from the corresponding author on reasonable request.

## Abbreviations

ANOVA: One-way analysis of variance
AT-II: Alveolar type II epithelial cells
BAL: Broncho-alveolar lavage
BALF: BAL fluid
CBPI: Cytokinesis-block proliferation index
DMPO: 5,5 Dimethyl-l-pyrroline-N-oxide
EPR: Electron paramagnetic resonance
FPG: *E. coli* formamidopyrimidine-DNA glycosylase
HIF: Hypoxia-inducible factor
hOGG1: human 8- oxoguanine DNA glycosylase 1
IARC: International Agency for Research on Cancer
LCO: LiCoO_2_
LDH: Lactate dehydrogenase
LIB: Li-ion battery
LTO: Li_4_Ti_5_O_12_
MN: Micronucleus
OH: Hydroxyl radical
PBS: Phosphate buffered saline
RLE: Rat lung epithelial cells
ROS: Reactive oxygen species
SEM: Standard error on the mean
TA: Terephthalate
WC-Co: Cobalt metal with tungsten carbide
WST-1: Water soluble tetrazolium salts 1
