# Supplementary material for: LiCoO2 particles used in Li-ion batteries induce primary mutagenicity in lung cells via their capacity to generate hydroxyl radicals
Source: Part Fibre Toxicol. 2020 Jan 29;17:6. doi: 10.1186/s12989-020-0338-9 (PMC6990559; doi:10.1186/s12989-020-0338-9)
Supplement: Supplementary file 1 — Additional file 1: Figure S1. LCO and LTO particle endocytosis by RLE cells. Rat lung epithelial cells (RLE) were exposed to NaCl or 50 μg/ml of LCO or LTO and treated with cytochalasin B 4 h after particle exposure. Endocytosis was assessed 24 h after particle exposure. Images of binucleated cells after treatment. White arrows designate some particles (a). One hundred binucleated cells were scored to determine the proportion of cells containing particles (b) as well as the number of endocytosed particles per cell (c). Bars represent means ± SEM (N = 1, n = 2). Figure S2. Inflammatory dose-response to LCO and LTO particles. Wistar rats were treated with an oro-pharyngeal aspiration of NaCl (control, CTL), 0.1, 0.3, 1 or 5 mg LCO or LTO particles. Inflammation was assessed after 3 d. LDH activity (a) and proteins (b) were measured in the BALF, recruited inflammatory cells in the BAL (c). Bars represent means ± SEM. *P < 0.05, **P < 0.01 and ***P < 0.001 relative to CTL mice (one-way ANOVA followed by a Dunnett’s multiple comparison, N = 1, n = 4). [file 12989_2020_338_MOESM1_ESM.pdf]

Particle and Fibre Toxicology

Additional file 1

$LiCoO_2$ PARTICLES USED IN LI-ION BATTERIES INDUCE PRIMARY MUTAGENICITY IN LUNG CELLS VIA THEIR CAPACITY TO GENERATE HYDROXYL RADICALS

Violaine Sironval§, Vittoria Scagliarini, Sivakumar Murugadoss, Maura Tomatis, Yousof Yakoub, Francesco Turci, Peter Hoet, Dominique Lison, Sybille van den Brule

§Corresponding author information:
Email address: violaine.sironval@uclouvain.be
Affiliation: Louvain centre for Toxicology and Applied Pharmacology, Institut de Recherche Expérimentale et Clinique, Université catholique de Louvain

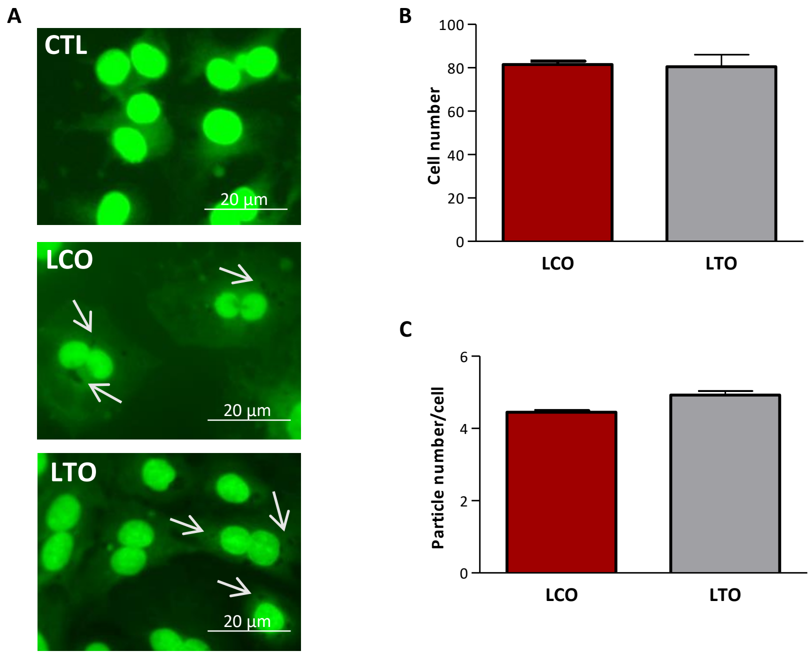

Figure 1: LCO and LTO particle endocytosis by RLE cells. Rat lung epithelial cells (RLE) were exposed to NaCl or 50 µg/ml of LCO or LTO and treated with cytochalasin B 4 h after particle exposure. Endocytosis was assessed 24 h after particle exposure. Images of binucleated cells after treatment. White arrows designate some particles **(a)**. 100 binucleated cells were scored to determine the proportion of cells containing particles **(b)** as well as the number of endocytosed particles per cell **(c)**. Bars represent means ± SEM (N = 1, n = 2).

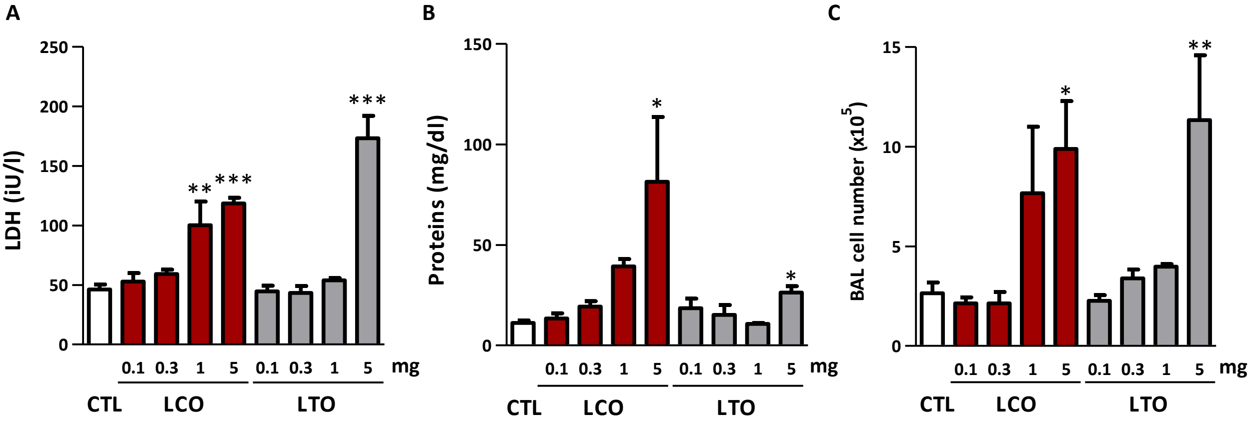

Figure 2: Inflammatory dose-response to LCO and LTO particles. Wistar rats were treated with an oro-pharyngeal aspiration of NaCl (control, CTL), 0.1, 0.3, 1 or 5 mg LCO or LTO particles. Inflammation was assessed after 3 d. LDH activity **(a)** and proteins **(b)** were measured in the BALF, recruited inflammatory cells in the BAL **(c)**. Bars represent means ± SEM. *P < 0.05, **P < 0.01 and ***P < 0.001 relative to CTL mice (one-way ANOVA followed by a Dunnett's multiple comparison, N = 1, n = 4).
